# Early inhalant allergen sensitization at component level: an analysis in atopic Dutch children

**DOI:** 10.3389/falgy.2023.1173540

**Published:** 2023-06-27

**Authors:** Lonneke J. Landzaat, Joyce A. M. Emons, Laura J. H. Sonneveld, Marco W. J. Schreurs, Nicolette J. T. Arends

**Affiliations:** ^1^ Division of Pediatric Respiratory Medicine and Allergology, Department of Pediatrics, Erasmus MC University Medical Center, Rotterdam, Netherlands; ^2^ Laboratory Medical Immunology, Department of Immunology, Erasmus MC University Medical Center, Rotterdam, Netherlands

**Keywords:** allergen, allergy, inhalant allergen, age group, sensitization, microarray analysis, rhinitis, allergen component.

## Abstract

**Background:**

Allergic rhinitis is a common respiratory disease in children and sensitization to inhalant allergens plays a significant role in its development. However, limited knowledge exists regarding sensitization profiles of inhalant allergen components in atopic children, particularly in the very young individuals. Understanding these profiles could provide insights into the early development of allergic rhinitis. The objective of this cross-sectional retrospective study was to evaluate the IgE-sensitization profiles to multiple inhalant allergen components and their clinical relevance in Dutch atopic children, with specific focus on children under the age of 4 years.

**Methods:**

A total of 243 atopic children were included in the study and sensitization profiles were analyzed using multiplex microarray analysis (ISAC). Clinical information was obtained from records of a pediatric allergy outpatient clinic between 2011 and 2020. Specific IgE responses to inhalation allergen components from five allergen sources (grass pollen, tree pollen, house dust mite, cat and dog), were examined. The study encompassed children of different age groups and compared those with and without symptoms.

**Results:**

The results demonstrated that sensitization to inhalant allergen components was present in 92% of the cohort. Sensitization was already evident at a young age (87%), including infancy, with a rapid increase in prevalence after 1 year of age. House dust mite emerged as the most predominant sensitizing allergen in early childhood, followed by tree pollen in later years. Sensitization patterns were similar between symptomatic and asymptomatic children, although symptomatic children exhibited higher frequencies and values. The sensitization profiles in very young children were comparable to those of children across all age groups.

**Conclusion:**

These findings highlight the presence of sensitization to inhalant allergen components and the early onset of allergic rhinitis before the age of 4, including infancy, in Dutch atopic children. Notable allergen molecules in Dutch atopic children under the age of 4 years include Bet v 1, Fel d 1, Der f 1, Der p 1, Der p 10 and Phl p 4, with house dust mite sensitization being the most common among Dutch infants. Moreover, the prevalence of sensitization to inhalant allergens in this Dutch cohort surpassed that of general European populations, emphasizing the importance of early assessment and management of allergic rhinitis in young atopic children.

## 1. Introduction

Allergic rhinitis is a common respiratory disease in children, with its prevalence increasing over time, ranging from 1 to 30% (1). However, limited data are available on the prevalence of allergic rhinitis in Dutch children. A Dutch population-based prospective cohort study, the Generation R Study, reported a prevalence of 12.4% for allergic rhinitis and 32.2% for inhalant sensitization at the age of 10 (2).

Atopic diseases often follow a characteristic sequence known as the “atopic march”, where atopic eczema and food allergy manifest in the early years of life, followed by respiratory allergies later on (3, 4) Previous studies have shown that sensitization to inhalant allergens typically occurs after infancy and increases thereafter (5–7). Sensitization to seasonal allergens (i.e., tree and grass pollen) tends to appear later than sensitization to perennial allergens (i.e., house dust mite) (5, 6).

In most studies, IgE antibody reactivity has been analyzed using singleplex assays with a limited number of allergen extracts. To gain a better understanding of allergic disease development, it is crucial to map the sensitization profiles of inhalant allergen components using advanced molecular allergology techniques, especially in very young children. Sensitization to different allergen components was found to be predictive of allergic disease development and treatment outcomes in children (8–11). Additionally, studies have shown similar sensitization profiles to inhalant allergen components in symptomatic and asymptomatic children (12). Therefore, obtaining more data on sensitization profiles in allergic populations, particularly in very young children, could provide valuable insights into the development and management of allergic rhinitis in early childhood.

The aim of this study was to assess the IgE sensitization profiles to multiple inhalant allergen components and their clinical relevance in Dutch atopic children, with a focus on the children under the age of 4 years.

## 2. Materials and methods

This cross-sectional retrospective study included 243 atopic children who were analyzed for presence of allergen specific IgE. The analysis was conducted using a multiplex microarray with 112 allergen components [Immuno-solid-phase Allergen Chip (ISAC), Thermo Fisher Scientific, Uppsala, Sweden]. The data collection period was between 2011 and 2020. Clinical information was obtained from our pediatric allergy outpatient clinic at the Erasmus MC Sophia Children's Hospital in Rotterdam, the Netherlands. The study included children who had been referred or were being treated by a pediatric allergist for a food allergy.

The study focused on 37 inhalant allergen components present on the ISAC. These components were derived from the five most significant sources of inhalation allergen: grass pollen, tree pollen, house dust mite, cat and dog. Table 1 provides an overview of the studied inhalation allergen components. Additionally, four allergen components (Pla a 2, Der p 23, Can f 4 and Can f 6) were added to the ISAC in 2017 and included in the analysis. Sensitization was considered present when the ISAC Standardized Units (ISU) value was equal or greater than 0.3.

**Table 1:** Overview inhalation allergen components in ISAC.

Grass pollen (*n* = 9)			Tree pollen (*n* = 12)					
**Name**	Bermuda grass	Timothy grass	Alder	Birch	Japanese cedar	Cypress	Olive tree	London plane tree
**Latin name**	Cynodon dactylon	Phleum pretense	Alnus glutinosa	Betula verrucose	Cryptomeria japonica	Cupressus arizonica	Olea europaea	Platanus acerifolia
**Allergen component name**	Cyn d 1	Phl p 1 Phl p 2 Phl p 4 Phl p 5 Phl p 6 Phl p 7 Phl p 11 Phl p 12	Aln g 1	Bet v 1 Bet v 2 Bet v 4	Cry j 1	Cup a 1	Ole e Ole e 7 Ole e 9	Pla a 1 Pla a 2 Pla a 3
House dust mite (*n* = 7)					Cat (*n* = 3)		Dog (*n* = 6)	
**Name**	American house dust mite	European house dust mite	Storage mite		Domestic cat		Domestic dog	
**Latin name**	Dermatophagoides farinae	Dermatophagoides pteronyssinus	Lepidoglyphus destructor		Felis domesticus		Canis familiaris	
**Allergen component name**	Der f 1 Der f 2	Der p 1 Der p 2 Der p 10 Der p 23	Lep d 2		Fel d 1 Fel d 2 Fel d 4		Can f 1 Can f 2 Can f 3 Can f 4 Can f 5 Can f 6	

For each patient, the medical records were retrospectively reviewed to identify symptoms of food allergy, rhinitis, asthma and/or eczema. Atopy was defined as having one or more of these diseases, accompanied by sensitization indicated by a skin prick test result larger than 4 mm or a specific IgE lever higher than 0.35 kU/liter. The diagnosis of food allergy required confirmation through a positive oral food challenge or a clear history of IgE-mediated symptoms such as angioedema, urticaria, dyspnea, rhinitis, gastro-intestinal and/or neurological symptoms after ingestion of the allergen. Symptoms related solely to oral allergy or sensitization to a food allergen without known clinical relevance were not classified as food allergies. Rhinitis was confimed when one or more symptoms of rhinorrhea, sneezing, nasal obstruction or nasal itching were present, with or without itchy, red or watery eyes. Asthma, including pre-school wheeze, was considered present if the patient was prescribed salbutamol and/or inhaled corticosteroids. Eczema was defined based on the use of topical corticosteroids. The presence of food allergy, rhinitis, asthma and eczema was also determined based on diagnoses mentioned in the medical chart by a pediatric allergist, pediatric pulmonologist and/or dermatologist.

### 2.1. Statistical analysis

The statistical analysis involved examining sensitization to inhalant components and symptoms of atopy across different age groups using an Analysis of Variance (ANOVA). The level of specific IgE for each positive allergen component was compared between symptomatic and asymptomatic children using the nonparametric U test. Statistical significance was defined as a *P*-value of ≤0.05. IBM SPSS Statistics was used for data analysis.

## 3. Results

### 3.1. Demographic and clinical characteristics of atopic children in different age groups

A total of 243 children (63% male, mean age 9.0 years) were included in this study. Table 2 presents the baseline characteristics for each age group. The majority of the children (88%) exhibited two or more atopic features, with food allergy being present in most cases (84%). Rhinitis was the most common symptom among older children, but it was already present in 55% of children under the age of 4. Asthma was diagnosed in 63% of the children, while eczema was present in the majority of children under 12 years (ranging from 84% to 90%). Among the cohort, 92% of the children, were sensitized to at least one of the 37 inhalant allergen components. Sensitization rates varied by age, with 87% in the youngest age group, increasing to 96% around the age of 12 to 15 years and then declining to 88%.

**Table 2:** Demographic and clinical characterization of atopic children.

	Total (All ages)	Group 1 (0–<4 year)	Group 2 (4–<8 year)	Group 3 (8–<12 year)	Group 4 (12–<15 year)	Group 5 (15–<18 year)
*N*	243	53	51	57	50	32
Male, *n* (%)	152 (63)	38 (72)	34 (67)	28 (49)	35 (70)	17 (53)
Female, *n* (%)	91 (37)	15 (28)	17 (33)	29 (51)	15 (30)	15 (47)
Age, years M ± SD	9.02 ± 5.00	2.46 ± 1.03	5.69 ± 1.19	9.83 ± 1.11	13.55 ± 0.93	16.69 ± 0.69
Age range, years	0.66–17.95	0.66–3.99	4.03–7.95	8.15–11.94	12.02–14.99	15.24–17.95
Rhinitis, *n* (%)	187 (77)	29 (55)	32 (63)	48 (84)	47 (94)	31 (97)
Asthma, *n* (%)	152 (63)	28 (53)	32 (63)	35 (61)	36 (72)	21 (66)
Eczema, *n* (%)	199 (82)	47 (89)	46 (90)	48 (84)	35 (70)	23 (72)
Food allergy, *n* (%)	203 (84)	47 (89)	44 (86)	44 (77)	43 (86)	25 (78)
Sensitization, *n* (%)	223 (92)	46 (87)	48 (94)	53 (93)	48 (96)	28 (88)

### 3.2. Sensitization to inhalant allergen components and symptoms are present at young age

**Figure 1:**
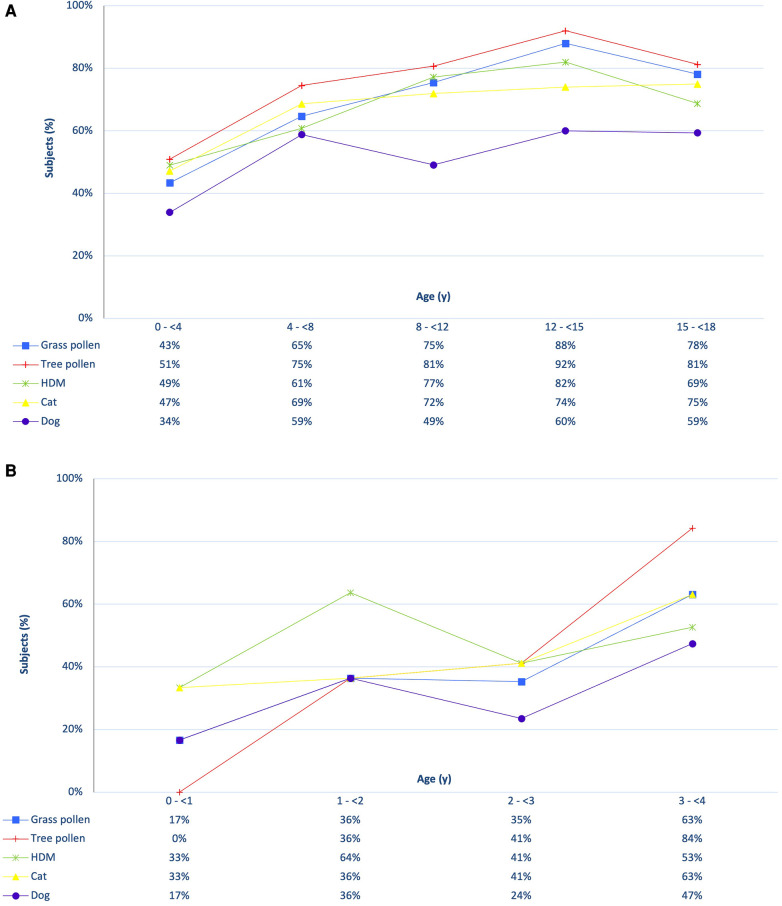
Presence of sensitization to inhalant allergens in all (**A**) and young (**B**) children.

Figure 1 provides an overview of sensitization to the five inhalant allergen sources divided by age category. Sensitization was already present in young children and increased rapidly after the age of 1 year (Figure 1B). In the first year of life, 50% of the casus exhibited sensitization to at least one of the 37 inhalation allergens, while in the subsequent three years these percentages were 91%, 88% and 95% respectively. House dust mite dominated sensitization in the first three years, after which tree pollen became the most common source. Figure 1A demonstrates that sensitization continues to increase during primary school, remaining stable or slightly decreasing afterwards. Among all sensitized children (*n* = 223), 80.7% had relevant sensitization with rhinitis symptoms, ranging from 57% in the youngest age group (*n* = 26) to 100% in the oldest age group (*n* = 28). In the 2 to 4 age group, the percentages of relevant sensitization with rhinitis were 67%, 89% and 98%, respectively, and these differences were statistically significantly (F = 12.41, *p* = 0.00); The likelihood of experiencing rhinitis symptoms when sensitized increased with age.

### 3.3. Similar sensitization patterns of inhalant allergen components, but less frequent present in asymptomatic children

**Figure 2:**
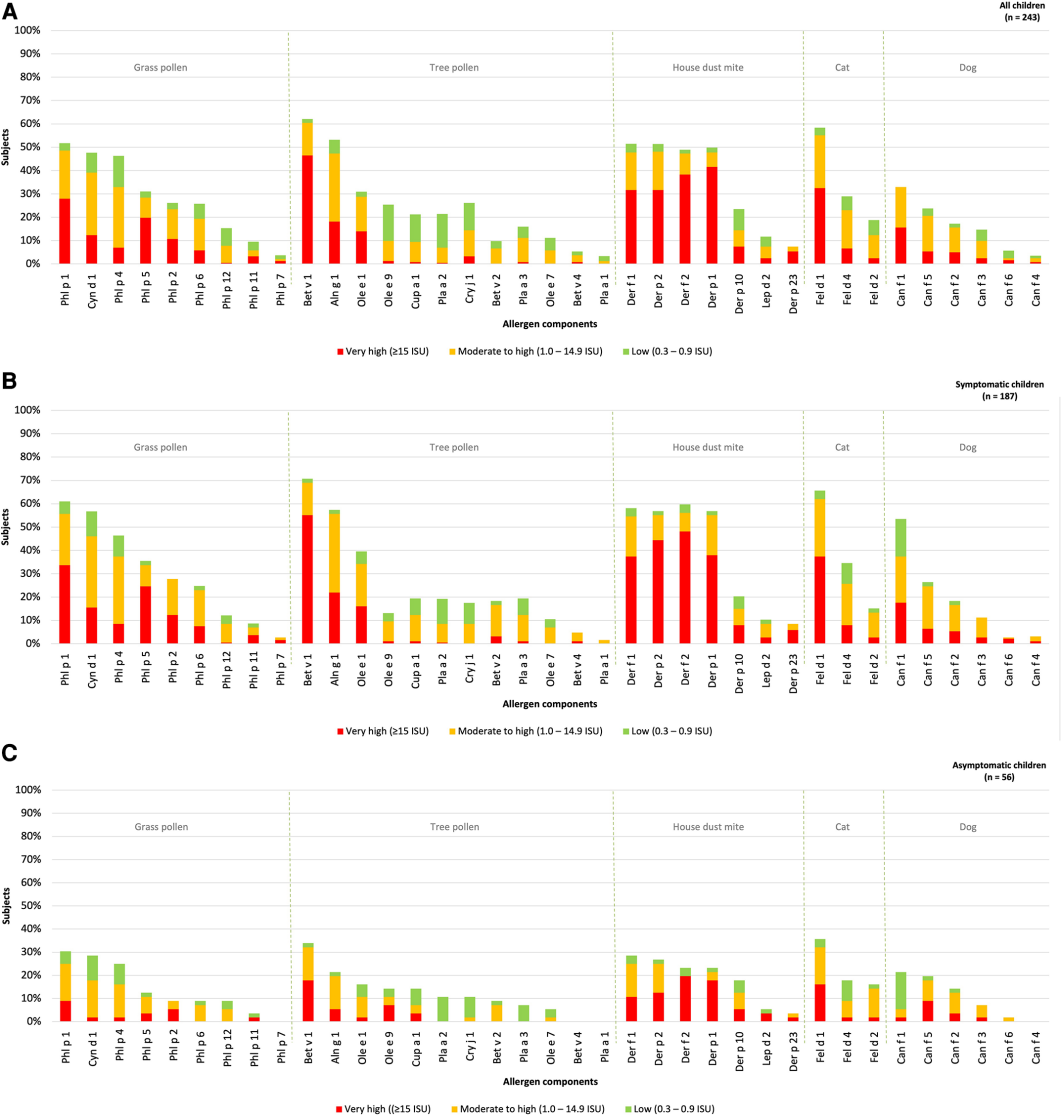
Sensitization patterns of inhalant allergen components in all (**A**), symptomatic (**B**) and asymptomatic (**C**) children.

The hierarchy of inhalant allergen components based on frequencies of specific IgE reactivity per inhalation group was determined for all children. The children were categorized into those with and without rhinitis symptoms (Figures 2A–C). The most frequently represented allergen components corresponded to the major allergens: Phl p 1 (52%) in grass pollen, Bet v 1 (62%) in tree pollen, Der f 1 (51%) and Der p 1 (51%) in house dust mite, Fel d 1 (58%) in cat and Can f 1 (33%) in dog. Sensitization to all 37 inhalation allergen components was observed in children with rhinitis (Figure 2B). In children without rhinitis, no sensitization was seen for Phl p 7, Bet v 4, Pla a 1 and Can f 4. The hierarchy of inhalant allergen components in symptomatic children was similar to that of asymptomatic children (Figures 2B,C). However, symptomatic children demonstrated higher frequencies of sensitization to Phl p 1 (59%), Bet v 1 (71%), Der f 1 (58%), Fel d 1 (65%) and Can f 1 (37%) compared to Phl p 1 (30%), Bet v 1 (34%), Der p 1 (29%), Fel d 1 (36%) and Can f 3 (21%) in asymptomatic children. As expected, the frequencies of sensitization were higher in symptomatic. Additionally, the levels of specific IgE were significantly higher for Phl p 1, Cyn d 1, Phl p 5, Bet v 1 and Can f 3 in symptomatic children (Supplementary Table S1A).

### 3.4. Sensitization patterns of inhalant allergen components in young atopic children

**Figure 3:**
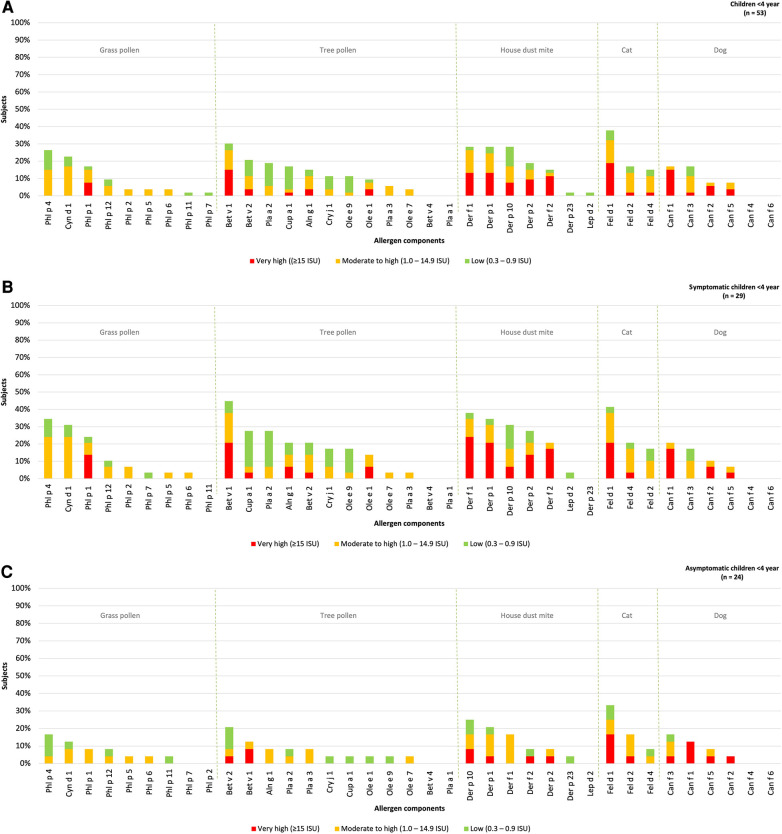
Sensitization patterns of inhalant allergen components in all (**A**), symptomatic (**B**) and asymptomatic (**C**) young children.

The hierarchy of inhalant allergen components based on the frequencies of specific IgE reactivity per inhalation group was determined for children under the age of 4 year (*n* = 53) and divided into those with or without rhinitis (Figure 3). Sensitization patterns in young children were similar to those observed in all children and were dominated by Phl p 4 (26%), Cyn d 1 (23%), Phl P 1 (17%), Bet v 1 (30%), Bet v 2 (21%), Def f 1, Derp 1, Der p 10 (all 28%), Fel d 1 (38%), Can f 1 and Can f 3 (both 17%). Differences were observed in the order of grass pollen components, with Phl p 4 and Cyn d 1 being more frequently represented in young, compared to Phl p 1 in older children. No sensitization was observed for Bet v 4, Pla a 1, Can f 4 and Can f 6, which aligns with the sensitization patterns in all children without symptoms.

The hierarchy inhalant allergen components in symptomatic children was found to be similar to that of asymptomatic children. The most dominant components in symptomatic children were Phl p 4 (34%), Bet v 1 (45%), Der f 1 (38%), Fel d 1 (41%) and Can f 1 (21%). These components were also observed as the most frequently recognized in asymptomatic children with Phl p 4 (17%), Bet v 2 (21%), Der p 10 (25%), Fel d 1 (33%), Can f 3 (17%) being the prominent ones (Figures 3B,C). Although there was no significant difference in the the levels of specific IgE for Phl p 1 and 4 between symptomatic and asymptomatic children, it was observed that 14% of the symptomatic children demonstrated a “very high” sensitization level to Phl p 1 compared to none of the asymptomatic children. Additionally, 24% of the symptomatic children showed a “moderate to high” sensitization level to Phl p 4, while only to 4% of asymptomatic children exhibited such sensitization. Bet v 2 and Can f 3 were predominantly present in asymptomatic children, but these components were also present at similar percentages in children with rhinitis (21% and 17% respectively). Overall, the frequency of sensitization was higher in symptomatic children, however the levels of specific IgE (measured in ISU) are not significantly higher in children with symptoms (Supplementary Table S1B).

## 4. Discussion

In this Dutch cohort of atopic pediatric patients, the prevalence of sensitization inhalant allergen components is 92%. This prevalence is higher compared to general European pediatric populations (25%–31%) (2, 13, 14) and more similar to other atopic cohorts (30%–100%) (5, 12, 15). Our findings indicate that IgE-reactivity to inhalant allergens is frequently present at very young age, with sensitization reported in half of the children under 1 year of age. The prevalence of sensitization rapidly increases prevalence from the year onwards and is clinically relevant in the majority of cases. Most studies on sensitization assess children in larger age groups or from the age of 4 years onwards, with limited data available on sensitization in atopic infants, which generally reports a lower prevalence (11%–30%) (5, 16, 17). The factors that predict allergic sensitization in the first year of life are still poorly understood. One possible explanation for the high number of sensitized young children in our cohort is the presence of eczema, as recent studies suggest that impaired skin barrier function in early infancy can lead to sensitization (4, 17, 18).

Although sensitization does not directly correspond to allergic symptoms, inhalant allergen sensitization is a significant factor in the development of allergic respiratory diseases. Early onset of inhalant sensitization (8, 9, 13, 14, 19), poly-sensitization (5, 20–22) and higher levels of specific IgE at young age (8, 14, 21, 22) have been associated with an increased risk of developing allergic rhinitis. Furthermore, sensitization to inhalant allergens during the first year of life appears to be a strong predictor for the development of allergic rhinitis later in life (19). This suggest that the young atopic sensitized children in our study who are asymptomatic will likely develop allergic rhinitis later in life, which aligns with the 100% prevalence of allergic rhinitis observed in our oldest children. Our results also indicate that allergic rhinitis can manifest earlier in life than previously thought. However, data on the prevalence of allergic rhinitis in infancy is limited and exhibits high variability due to the lack of universal criteria (17, 23, 24). Allergic rhinitis, generally considered as a mild disease, carries a significant burden that is often underestimated. Studies have revealed that allergic rhinitis adversely affects children's quality of life, sleep patterns, school and physical and emotional well-being (25). Therefore, it is crucial to consider and recognize allergic rhinitis in atopic children from a very young age.

Although house dust mite sensitization dominates in the first three years of life, tree pollen sensitization becomes more prevalent in adulthood. This confirms previous findings that identify house dust mite as the main sensitizing inhalant allergens in early life. House dust mite sensitization has been reported to be most prevalent in atopic infants from Italy (40%), Sweden (11%), Belgium (11%) and Japan (31%) (5, 16, 17, 24) and is associated with presence or development of allergic respiratory disease (17, 19, 23, 24). Other studies have reported sensitization to grass and tree pollen in Dutch children at rates of 16% and 11% respectively, with house dust mite sensitization (20%) including allergic symptoms being the most dominant at 10 years of age (2). Similar results have been found in the Swedish BAMSE-cohort and German MAS-cohort were tree and grass pollen sensitization become more common in later life (13, 14).

This is the first study to establish specific molecular IgE sensitization profiles in Dutch atopic children. While the rates of sensitization to inhalant allergen components may vary between regions due to differences in allergen exposure, our study reveals a profile characterized by dominant sensitization of Bet v 1, Fel d 1, Phl p 1 and various house dust mite components, which is consistent with findings in other European children (9, 12). Notably, major allergens associated with house dust mites include Der p 1, Der p 2, Der f 1, and Der f 2. Nevertheless, there exists heterogeneity in reported sensitization to Der p 10 across Europe, with children showing relatively high levels of sensitization, similar to our findings (26). Symptomatic and asymptomatic children exhibited similar sensitization patterns to allergen molecules and symptoms were associated with higher frequencies and IgE-levels of major allergen components such as Bet v 1 and Phl p 1. In young children, Fel d 1 and Bet v 1 were also prominent allergen components, along with Der f 1, Der p 1, Der p 10 and Phl p 4. The presence of specific allergen components during early childhood has been identified as a predictive factor for the development of respiratory diseases later in life. Previous studies in several Northern European countries have demonstrated the association between sensitization to allergens such as Fel d 1, Phl p 1, Bet v 1, Der p 1 and Der f 2 and the subsequent onset of respiratory conditions (9, 20, 27). Additionally, elevated Bet v 1-specific IgE during early age have been linked to more severe rhinitis symptoms in adolescence (8). Moreover, Phl p 4 has been identified as an early indicator of grass pollen allergy, along with Phl p 1 (10), implying young children in our cohort will likely develop grass pollen allergy later in life.

This study has some limitations. It is a cross-sectional analysis, lacking longitudinal follow-up of patients and the analysis is conducted and compared across different age groups at a single time point. Therefore, the progression and potential remission of sensitization cannot be determined from this analysis alone. It is important to note that the study cohort consists of allergic patients who were primarily diagnosed and treated at the pediatric allergy outpatient clinic. Thus, caution must be exercised when extrapolating these findings to the general pediatric population.

In conclusion, this study is first to provide insights into sensitization patterns at allergen molecular level in Dutch atopic children, highlighting the presence of sensitization to inhalant allergen components and associated symptoms before the age of 4 year, including infancy. It may indicate that sensitization to inhalant allergen components is shifting towards an earlier onset in atopic children, challenging the notion that the prevalence of allergic rhinitis primarily increases during primary school years as commonly indicated in literature but can also manifest during infancy. Furthermore, Bet v 1, Fel d 1, Der f 1, Der p 1, Der p 10 and Phl p 4 are identified as prominent allergen molecules in Dutch atopic children under the age of 4 year, while and house dust mite sensitization is most prevalent among Dutch infants. Early diagnosis of allergic rhinitis is crucial to initiate timely treatment and alleviate the associated burden. This necessitates early determination of specific IgE to inhalant allergens, which can be performed as early as infancy. Additionally, gaining further understanding of sensitization prevention strategies, such as improving skin barrier function, and exploring treatment opportunities, such as immunotherapy in sensitized atopic children under the age of 4 would be of great interest. Consequently, more studies focusing on young atopic children are necessary to expend our knowledge in this aera.

## Data Availability

The raw data supporting the conclusions of this article will be made available by the authors, without undue reservation.
