# Supplementary material for: Early inhalant allergen sensitization at component level: an analysis in atopic Dutch children
Source: Front Allergy. 2023 Jun 27;4:1173540. doi: 10.3389/falgy.2023.1173540 (PMC10352100; doi:10.3389/falgy.2023.1173540)
Supplement: Supplementary file 1 [file Table1.docx]

*Supplementary Materials*

Table 1 Sensitization levels and frequencies of inhalant allergen components in A) all atopic children and B) young atopic children

A

| Allergens | Symptomatic children,  n = 187 | | Asymptomatic children,  n = 57 | | Specific IgE symptomatic vs asymptomatic, p values |
| --- | --- | --- | --- | --- | --- |
|  | ISU†, Median  [Q1; Q3] | Numbers of positive subjects, n (%) | ISU†, Median  [Q1; Q3] | Numbers of positive subjects, n (%) |  |
| *Grass pollen* | | | | | |
| Phl p 1 | 21.8 [4.8; 54.8] | 110 (58.8) | 9.3 [2.4; 20.2] | 17 (29.8) | 0.018* |
| Cyn d 1 | 6.8 [1.9; 19.3] | 102 (54.5) | 3.5 [0.8; 4.7] | 14 (24.6) | 0.006* |
| Phl p 4 | 3.7 [1.0; 11.0] | 95 (50.8) | 1.3 [0.6; 6.6] | 16 (28.1) | 0.139 |
| Phl p 5 | 35.2 [8.4; 72.5] | 68 (36.4) | 11.5 [4.5; 15.7] | 7 (12.3) | 0.035* |
| Phl p 2 | 9.2 [2.5; 23.2] | 57 (30.5) | 16.9 [5.3; 31.2] | 5 (8.8) | 0.453 |
| Phl p 6 | 11.0 [1.7; 15.4] | 55 (29.4) | 4.5 [1.0; 7.4] | 5 (8.8) | 0.659 |
| Phl p 12 | 1.1 [0.5; 4.9] | 30 (16.0) | 2.0 [0.4; 6.1] | 5 (8.8) | 1.000 |
| Phl p 11 | 8.5 [0.4; 17.4] | 20 (10.7) | 10.1 [0.7; 19.5] | 2 (3.5) | 0.819 |
| Phl p 7 | 3.1 [0.5; 33.7] | 8 (4.3) | 0 | 0 | n.a. |
| *Tree pollen* | | | | | |
| Bet v 1 | 43.8 [18.2; 96.1] | 132 (70.6) | 21.6 [4.0; 33.1] | 19 (33.3) | 0.004* |
| Aln g 1 | 9.1 [3.2; 25.2] | 115 (61.5) | 6.3 [2.7; 15.6] | 12 (21.1) | 0.589 |
| Ole e 1 | 12.3 [4.1; 32.3] | 68 (36.4) | 12.5 [1.0; 38.7] | 8 (14.0) | 0.648 |
| Ole e 9 | 0.9 [0.6; 3.0] | 47 (25.1) | 1.5 [0.6; 5.2] | 9 (15.8) | 0.372 |
| Cup a 1 | 1.0 [0.6; 2.9] | 45 (24.1) | 0.6 [0.5; 0.8] | 6 (10.5) | 0.132 |
| Pla a 2 | 0.79 [0.5; 1.7] | 43 (23.0) | 0.8 [0.4; 1.0] | 6 (10.5) | 0.376 |
| Cry j 1 | 0.9 [0.5; 1.8] | 38 (20.3) | 0.5 [0.4; 0.7] | 4 (7.0) | 0.123 |
| Bet v 2 | 3.3 [1.5; 11.0] | 37 (19.8) | 1.0 [0.4; 15.9] | 8 (14.0) | 0.138 |
| Pla a 3 | 2.4 [0.7; 6.4] | 32 (17.1) | 1.2 [0.9; 4.5] | 5 (8.8) | 0.534 |
| Ole e 7 | 1.3 [0.5; 3.1] | 23 (12.3) | 0.7 [0.5; 4.8] | 3 (5.3) | 0.872 |
| Bet v 4 | 4.0 [1.0; 11.6] | 12 (6.4) | 0 | 0 | n.a. |
| Pla a 1 | 0.5 [0.4; 2.2] | 7 (3.7) | 0 | 0 | n.a. |
| *HDM* | | | | | |
| Der f 1 | 26.3 [9.7; 57.1] | 109 (58.3) | 10.4 [2.0; 41.5] | 15 (26.3) | 0.110 |
| Der p 2 | 45.1 [19.0; 89.6] | 109 (58.3) | 50.8 [9.1; 149.4] | 13 (22.8) | 0.627 |
| Der f 2 | 53.8 [21.7; 97.0] | 108 (57.8) | 58.9 [15.4; 135.9] | 13 (22.8) | 0.694 |
| Der p 1 | 32.3 [8.2; 79.8] | 107 (57.2) | 12.0 [2.7; 69.8] | 16 (28.1) | 0.160 |
| Der p 10 | 2.7 [0.8; 19.8] | 45 (24.1) | 2.1 [0.7; 25.9] | 10 (17.5) | 0.887 |
| Lep d 2 | 1.5 [0.8; 5.9] | 24 (12.8) | 14.8 [7.2; 22.4] | 2 (3.5) | 0.124 |
| Der p 23 | 33.4 [9.6; 73.1] | 16 (8.6) | 15.4 [0.3; 50.6] | 3 (5.3) | 0.314 |
| *Cat* | | | | | |
| Fel d 1 | 19.3 [7.4; 55.9] | 122 (65.2) | 13.6 [3.6; 52.2] | 20 (35.1) | 0.323 |
| Fel d 4 | 3.7 [1.2; 15.1] | 59 (31.6) | 2.8 [1.9; 7.0] | 9 (15.8) | 0.711 |
| Fel d 2 | 1.5 [0.6; 6.4] | 37 (19.8) | 0.9 [0.5; 2.4] | 10 (17.5) | 0.275 |
| *Dog* | | | | | |
| Can f 1 | 14.5 [5.6; 39.5] | 70 (37.4) | 13.9 [6.9; 26.9] | 11 (19.3) | 0.416 |
| Can f 5 | 4.9 [1.7; 12.5] | 52 (27.8) | 2.9 [1.6; 32.7] | 4 (7.0) | 0.750 |
| Can f 2 | 6.9 [2.9; 21.7] | 34 (18.2) | 2.7 [1.3; 19.4] | 8 (14.0) | 0.179 |
| Can f 3 | 2.4 [0.7; 9.0] | 30 (16.0) | 0.7 [0.4; 1.0] | 12 (21.1) | 0.024* |
| Can f 6 | 0.9 [0.5; 105.0] | 11 (5.9) | 4.32 [-;-] | 1 (1.8) | 0.664 |
| Can f 4 | 2.1 [0.9; 109.9] | 8 (4.3) | 0 | 0 | n.a. |

B

| Allergens | Symptomatic young children (<4y),  n = 29 | | Asymptomatic young children (<4y),  n = 24 | | Specific IgE symptomatic vs asymptomatic, p values |
| --- | --- | --- | --- | --- | --- |
|  | ISU†, Median  [Q1; Q3] | Numbers of positive subjects, n (%) | ISU†, Median  [Q1; Q3] | Numbers of positive subjects, n (%) |  |
| *Grass pollen* | | | | | |
| Phl p 1 | 18.9 [1.1; 28.6] | 7 (24.1] | 4.0 [2.7; 5.3] | 2 (8.3) | 0.558 |
| Cyn d 1 | 1.8 [1.0; 5.8] | 9 (31.0) | 1.08 [0.3; 3.7] | 3 (12.5) | 0.229 |
| Phl p 4 | 1.4 [0.6; 2.2] | 10 (34.5) | 0.8 [0.5; 3.3] | 4 (16.7) | 0.480 |
| Phl p 5 | 7.9 [-; -] | 1 (3.5) | 6.1 [-; -] | 1 (4.2) | 0.317 |
| Phl p 2 | 7.3 [4.7; 9.9] | 2 (6.9) | 0 | 0 | n.a. |
| Phl p 6 | 1.85 [-; -] | 1 (3.5) | 1.0 [-; -] | 1 (4.2) | 0.317 |
| Phl p 12 | 1.0 [0.8; 1.7] | 3 (10.3) | 1.3 [0.5; 2.0] | 2 (8.3) | 1.000 |
| Phl p 11 | 0 | 0 | 0.7 [-; -] | 1 (4.2) | n.a. |
| Phl p 7 | 0.8 [-; -] | 1 (3.5) | 0 | 0 | n.a. |
| *Tree pollen* | | | | | |
| Bet v 1 | 14.8 [1.4; 57.7] | 13 (44.8) | 33.1 [4.0; 57.7] | 3 (12.5) | 0.459 |
| Aln g 1 | 8.3 [0.6; 17.6] | 6 (20.7) | 9.9 [6.3; 13.5] | 2 (8.3) | 1.000 |
| Ole e 1 | 11.4 [1.4; 31.9] | 4 (13.8) | 0.5 [-; -] | 1 (4.2) | 0.157 |
| Ole e 9 | 0.4 [0.3; 2.3] | 5 (17.2) | 0.7 [-; -] | 1 (4.2) | 0.380 |
| Cup a 1 | 0.7 [0.6; 10.0] | 8 (27.6) | 0.7 [-; -] | 1 (4.2) | 0.437 |
| Pla a 2 | 0.7 [0.4; 2.7] | 8 (27.6) | 1. [0.8; 1.1] | 2 (8.3) | 0.295 |
| Cry j 1 | 0.6 [0.5; 6.7] | 5 (17.2) | 0.7 [-; -] | 1 (4.2) | 0.770 |
| Bet v 2 | 2.3 [0.5; 8.3] | 6 (20.7) | 0.4 [0.4; 11.2] | 5 (20.8) | 0.465 |
| Pla a 3 | 2.7 [-; -] | 1 (3.5) | 2.7 [1.2; 4.2] | 2 (8.3) | 1.000 |
| Ole e 7 | 1.4 [-; -] | 1 (3.5) | 4.8 [-; -] | 1 (4.2) | 0.317 |
| Bet v 4 | 0 | 0 | 0 | 0 | n.a. |
| Pla a 1 | 0 | 0 | 0 | 0 | n.a. |
| *HDM* | | | | | |
| Der f 1 | 17.9 [1.5; 36.2] | 11 (37.9) | 2.9 [1.5; 8.8] | 4 (16.7) | 0.151 |
| Der p 2 | 18.2 [1.7; 83.5] | 8 (27.6) | 13.1 [1.2; 25.1] | 2 (8.3) | 0.794 |
| Der f 2 | 41.9 [23.4; 88.7] | 6 (20.7) | 8.2 [0.8; 15.5] | 2 (8.3) | 0.096 |
| Der p 1 | 28.6 [1.7; 48.9] | 10 (34.5) | 2.6 [1.4; 64.1] | 5 (20.8) | 0.540 |
| Der p 10 | 1.7 [0.6; 33.0] | 9 (31.0) | 4.3 [0.7; 25.9] | 6 (25.0) | 0.814 |
| Lep d 2 | 0.5 [-; -] | 1 (3.5) | 0 | 0 | n.a. |
| Der p 23 | 0 | 0 | 0.3 [-; -] | 1 (4.2) | n.a. |
| *Cat* | | | | | |
| Fel d 1 | 15.4 [1.1; 84.3] | 12 (41.4) | 16.7 [1.1; 84.3] | 8 (33.3) | 1.000 |
| Fel d 4 | 1.9 [1.2; 8.1] | 6 (20.7) | 2.1 [0.5; 3.6] | 2 (8.3) | 0.739 |
| Fel d 2 | 1.2 [0.5; 4.2] | 5 (17.2) | 3.5 [1.2; 81.2] | 4 (16.7) | 0.268 |
| *Dog* | | | | | |
| Can f 1 | 35.7 [13.4; 57.9] | 6 (20.7) | 24.4 [23.1; 34.6] | 3 (12.5) | 0.606 |
| Can f 5 | 10.6 [5.0; 16.1] | 2 (6.9) | 22.0 [1.7; 42.3] | 2 (8.3) | 1.000 |
| Can f 2 | 35.8 [1.5; 50.1] | 3 (10.3) | 24.6 [-; -] | 1 (4.2) | 0.655 |
| Can f 3 | 1.5 [0.5; 3.3] | 5 (17.2) | 4.7 [0.9; 16.6] | 4 (16.7) | 0.327 |
| Can f 6 | 0 | 0 | 0 | 0 | n.a. |
| Can f 4 | 0 | 0 | 0 | 0 | n.a. |

†ISU, ISAC standardized units. Reference values: <0,3 ISU negative; 0,3-0,9 ISU low; 1-14,9 ISU moderate to high; ≥15 ISU very high
*p ≤0.05
